# Prevalence and determinants of anemia in chronic kidney disease patients in Ethiopia: a systematic review and meta-analysis

**DOI:** 10.3389/fmed.2025.1529280

**Published:** 2025-06-27

**Authors:** Eshetu Elfios Endrias, Temesgen Geta, Eskinder Israel, Melesse Belayneh Yayeh, Beker Ahmed, Abdulkerim Hassen Moloro

**Affiliations:** ^1^School of Nursing, College of Health Science and Medicine, Wolaita Sodo University, Wolaita Sodo, Ethiopia; ^2^School of Public Health, College of Health Science and Medicine, Wolaita Sodo University, Wolaita Sodo, Ethiopia; ^3^Department of Public Health, School of Public Health, College of Medicine and Health Science, Bahir Dar University, Bahir Dar, Ethiopia; ^4^Department of Midwifery, College of Health Science and Medicine, Arsi University, Asela, Ethiopia; ^5^Department of Nursing, College of Medicine and Health Science, Samara University, Samara, Ethiopia

**Keywords:** chronic kidney disease, CKD, anemia prevalence, diabetes mellitus, systematic review, meta-analysis

## Abstract

**Background:**

Chronic kidney disease (CKD) is a progressive and devastating condition affecting over 10% of the global population, with more than 800 million individuals impacted worldwide. Anemia, a critical and debilitating complication of CKD, accelerates disease progression and exacerbates its overall burden. Despite numerous studies exploring the prevalence and determinants of anemia in CKD in Ethiopia, the pooled prevalence remains undefined, highlighting a critical gap in evidence.

**Objectives:**

To determine the pooled prevalence of anemia and identify its associated factors among CKD patients in Ethiopia through a systematic review and meta-analysis.

**Methods:**

A comprehensive search was carried out for studies with full document and written in English language through an electronic web-based search strategy from databases of PubMed, CINAHL, Cochrane Library, Embase, Google Scholar and Ethiopian University Repository online. The quality of the included studies was assessed using the Joanna Briggs Institute (JBI) checklist. Statistical analyses were performed using STATA version 17. Meta-analysis was conducted with a random-effects model. Heterogeneity among the primary studies was evaluated using Cochran’s Q test and the I-squared statistic. To explore potential sources of heterogeneity, subgroup and sensitivity analyses were performed.

**Results:**

This systematic review and meta-analysis included seven studies, involving a total of 1,714 CKD patients. The pooled prevalence of anemia among CKD patients was 60.93% (95% CI: 50.05, 71.80), with substantial heterogeneity observed across studies (I^2^ = 97.0, *P* < 0.000). A significant association was found between anemia in CKD patients and diabetes comorbidity, with those having diabetes experiencing significantly higher odds of developing anemia (OR: 4.52, 95% CI: 1.18–7.28). Additionally, patients in stage 3 or more advanced stages of CKD had an increased risk of anemia (OR: 4.36, 95% CI: 3.15–5.57).

**Conclusion and recommendations:**

This systematic review and meta-analysis revealed a high pooled prevalence of anemia among CKD patients, with significant associations with diabetes mellitus comorbidity, and advanced CKD stages. Regional differences were also noted, with Oromia region showing the highest prevalence. We recommend regular anemia screening in CKD patients and the identification and management of risk factors such as diabetes and advanced CKD stages.

**Systematic review registration:**

https://www.crd.york.ac.uk/PROSPERO/view/CRD42024608561, identifier CRD42024608561.

## Background

Chronic kidney disease (CKD) is characterized by persistent abnormalities in kidney structure or function lasting at least 3 months, with significant health implications. CKD is classified according to Cause, Glomerular Filtration Rate (GFR) category (G1–G5), and Albuminuria category (A1–A3), collectively referred to as the CGA classification (1). CKD is a progressive disease that affects over 10% of the global population, impacting more than 800 million people (2). According to the Global Burden of Disease studies, CKD has become a major cause of mortality worldwide (3, 4).

According to the World Health Organization (WHO), anemia is a significant public health issue worldwide (5). It is defined as hemoglobin (Hgb) concentration of less than 12 g/dL for females and less than 13 g/dL for males (6, 7). Anemia is a significant complication of CKD, accelerating disease progression and increasing its overall health burden (8). Anemia prevalence is notably elevated among CKD patients ranging from 14% to 91% (9–13).

Anemia associated with CKD, sometimes referred to as anemia of chronic renal disease, is classified as normocytic normochromic anemia and is characterized by a reduced ability to produce red blood cells. This form of anemia is frequently observed in individuals with renal impairment and is correlated with poor health outcomes, including an increased risk of mortality among CKD patients (14). Anemia in CKD is characterized by hemoglobin levels that fall below the average range adjusted for age and sex. The primary cause of this anemia is the insufficient production of endogenous erythropoietin, which can result from either functional or absolute deficiencies of iron (15).

Various factors contribute to the development of anemia in individuals with chronic kidney disease (CKD), including diabetic nephropathy, the stage of CKD, body mass index (BMI), smoking status, white blood cell (WBC) count, and serum albumin levels (16). As CKD progresses, hemoglobin levels decline primarily due to the insufficient production of erythropoietin by impaired kidneys. Furthermore, anemia is exacerbated by hepcidin, which limits iron availability by reducing both intestinal iron absorption and the mobilization of stored iron needed for red blood cell production (17).

Anemia is linked to reduced quality of life (QoL) and increased rates of cardiovascular disease and death (18). A study which enrolled 27,998 patients with CKD and followed them for nearly 5.5 years, showed that the prevalence rates of anemia, congestive heart failure, coronary artery disease, and type 2 diabetes mellitus were higher among patients who died compared to those who survived, despite a shorter observation period for the deceased (19).

Erythropoiesis-stimulating agents (ESAs) and iron have been integral in the management of anemia related to CKD for many years (14, 20). Recent developments have introduced innovative strategies for enhancing erythropoiesis, including novel iron formulations. One of the most promising methods involves targeting the hypoxia-inducible transcription factor (HIF) pathway. Additionally, the modulation of the activin A signaling pathway is gaining attention for its potential benefits, which include increased bone density. Furthermore, newly formulated intravenous iron products may offer the advantage of fewer required administration sessions (21).

While previous study have examined the prevalence and associated factors of anemia among CKD patients in Sub-Saharan Africa (8), there remains a critical need for an Ethiopia-specific analysis. Regional and global studies provide valuable insights but may overlook Ethiopia’s unique demographic, cultural, and healthcare dynamics, which likely influence anemia prevalence and risk factors differently from other parts of globe and Sub-Saharan Africa. Given these contextual differences, synthesizing data specifically from Ethiopian studies will allow for more relevant and precise recommendations for anemia management in CKD patients within the country. This focused review will support policymakers and healthcare providers in designing targeted, culturally appropriate interventions to address the unique needs of Ethiopia’s CKD population. Therefore, this study aims to address the following research questions:

1. What is the pooled prevalence of anemia among CKD patients in Ethiopia?
2. What are the factors associated with anemia among CKD patients in Ethiopia?

### Objectives

The primary objective of this study is to determine the pooled prevalence of anemia among CKD patients in Ethiopia. The secondary objective is to identify the factors associated with anemia in this population.

## Methods

### Study design and search strategy

A comprehensive systematic review and meta-analysis were conducted to investigate the prevalence and determinants of anemia in CKD patients in Ethiopia. The methodology for this systematic review and meta-analysis was developed in accordance with the Preferred Reporting Items for Systematic Reviews and Meta-Analyses Protocols (PRISMA-P) statement (22). The findings were reported following the PRISMA guidelines (23).

We performed a systematic and comprehensive search across multiple databases, including PubMed, CINAHL, Cochrane Library, Embase, Google Scholar, and the Ethiopian University Repository, to identify studies reporting on anemia among CKD patients in Ethiopia. To ensure no duplication of efforts, we reviewed the database available at http://www.library.ucsf.edu and the Cochrane Library. Additionally, we screened reference lists of relevant articles to retrieve further studies. The process involved utilizing EndNote (version X8) software for downloading, organizing, reviewing, and citing articles. A manual search for cross-references was also conducted to find any pertinent studies that may not have been captured in the initial database search.

The search utilized a comprehensive set of keywords, including “prevalence,” “anemia,” “chronic kidney disease,” “CKD,” “Ethiopian patients,” “renal disease,” and “Ethiopia.” These terms were combined using Boolean operators (AND, OR) to facilitate a thorough and systematic search across the specified databases.

### Eligibility criteria

Inclusion Criteria: The following criteria were established to guide the selection of articles for this meta-analysis and systematic review:

1. Participants: Studies must include individuals living in Ethiopia.
2. Type of Research: Only community or institutional-based studies conducted in Ethiopia were eligible.
3. Study Design: All observational study designs, such as cross-sectional, case-control, and cohort studies, were considered for inclusion.
4. Setting: Studies had to be conducted in Ethiopia.
5. Study Format: Publishedworks, were included up until the final date of data analysis.
6. Language: This review only considered studies published in English.

Exclusion Criteria: Studies were excluded if they lacked full text or demonstrated poor methodological quality. Research which scored Joanna Briggs Institute (JBI) quality index score of seven or above were categorized as low risk and below were considered as poor methodological quality. Additionally, studies that did not provide information on anemia in CKD patients or for which necessary information could not be obtained were also excluded. Three authors (E.E., B.A., M.B.) independently assessed the eligibility of the identified studies, with input from another author (E.I) to reach a consensus on potential inclusion or exclusion.

### Quality assessment

Three authors (A.H and T.G) independently performed a critical appraisal of the studies included in this review, utilizing the Joanna Briggs Institute (JBI) checklist for prevalence studies to evaluate quality. This tool comprises nine criteria with options for responses: yes, no, unclear, and not applicable (24). Additionally, reviewer E.E provided input as needed during the assessment process. Studies scoring seven or higher on the Newcastle–Ottawa Quality Assessment Scale (NOS) were deemed of acceptable quality (24). Consequently, all studies were included in our review (Table 1).

**TABLE 1:** Critical appraisal results of eligible studies on prevalence of anemia and its associated factors among chronic kidney disease (CKD) patients, Ethiopia, 2024.

Name of author	Q1	Q2	Q3	Q4	Q5	Q6	Q7	Q8	Q9	Total
F Bishaw et al.	Y	Y	Y	N	Y	Y	Y	Y	Y	8
B. Alemu et al.	Y	Y	Y	Y	Y	Y	Y	Y	Y	9
H. Adera et al.	Y	N	Y	Y	Y	N	Y	Y	Y	7
T H Molla et al	Y	Y	Y	Y	Y	Y	Y	Y	Y	9
A Kidanewold et al	Y	Y	Y	Y	Y	Y	Y	Y	Y	9
M U Yusuf et al	Y	Y	Y	Y	Y	Y	Y	Y	Y	9
S Abdi et al	Y	Y	Y	N	Y	Y	Y	N	Y	7

Y, yes; N,no; JBI critical appraisal checklist for studies reporting prevalence data: Q1 = was the sample frame appropriate to address the target population? Q2-Were study participants sampled appropriately? Q3-Was the sample size adequate? Q4-Were the study subjects and the setting described in detail? Q5-Was the data analysis conducted with sufficient coverage of the identified sample. Q6-Were the valid methods used for the identification of the condition? Q7-Was the condition measured in a standard, reliable way for all participants? Q8-Was there appropriate statistical analysis? Q9-Was the response rate adequate, and if not, was the low response rate managed appropriately?

The methodological quality of studies examining anemia in CKD patients and its associated factors in Ethiopia was evaluated based on the following questions:

Was the sample frame appropriate to address the target population?

Were study participants sampled appropriately?

Was the sample size adequate?

Were the study subjects and setting described in detail?

Was data analysis conducted with sufficient coverage of the identified sample?

Were valid methods used to identify the condition?

Was the condition measured in a standardized, reliable way for all participants?

Was appropriate statistical analysis used?

Was the response rate adequate, and if not, was a low response rate managed appropriately?

### Data extraction

Data was extracted and documented using Microsoft Excel, guided by the Joanna Briggs Institute (JBI) data extraction form for observational studies. Two authors (E.E., B.A) independently conducted the data extraction process. The recorded data included the first author’s last name, publication year, study setting or country, region, study design, study period, sample size, response rate, population characteristics, types of management, prevalence of anemia, and associated factors. Any discrepancies in the data extraction were resolved through discussion among the extractors.

### PICO components for anemia prevalence among CKD patients

The PICO statement for our review highlights the key components of the research design. The population of interest consists of patients with CKD. The intervention focuses on studies investigating the prevalence of anemia within this population. The comparative aspect involves examining studies that report anemia prevalence among CKD patients in different regions or settings. The primary outcome of interest is the prevalence of anemia observed among CKD patients. Additionally, the secondary outcome seeks to identify the factors associated with anemia in this population.

### Heterogeneity and publication bias

To assess potential publication bias and the effects of small studies, we utilized funnel plots along with Egger’s test. Furthermore, we calculated the I^2^ statistic to evaluate heterogeneity among the studies included in the analysis (25, 26).

### Data processing and analysis

Data analysis involved importing the extracted data into STATA 14 statistical software to calculate the pooled prevalence of anemia among CKD patients in Ethiopia. A meta-analysis was performed using a random-effects model to account for variability across studies (27). Subgroup analyses were conducted to compare the prevalence of anemia and its associated factors among CKD patients across different regions of Ethiopia. The results were visually presented in a forest plot format with a 95% confidence interval. I^2^ values of 0%, 25%, 50%, and 75% were interpreted as indicating no, low, medium, and high heterogeneity, respectively (28).

## Results

### Study selection

**FIGURE 1:**
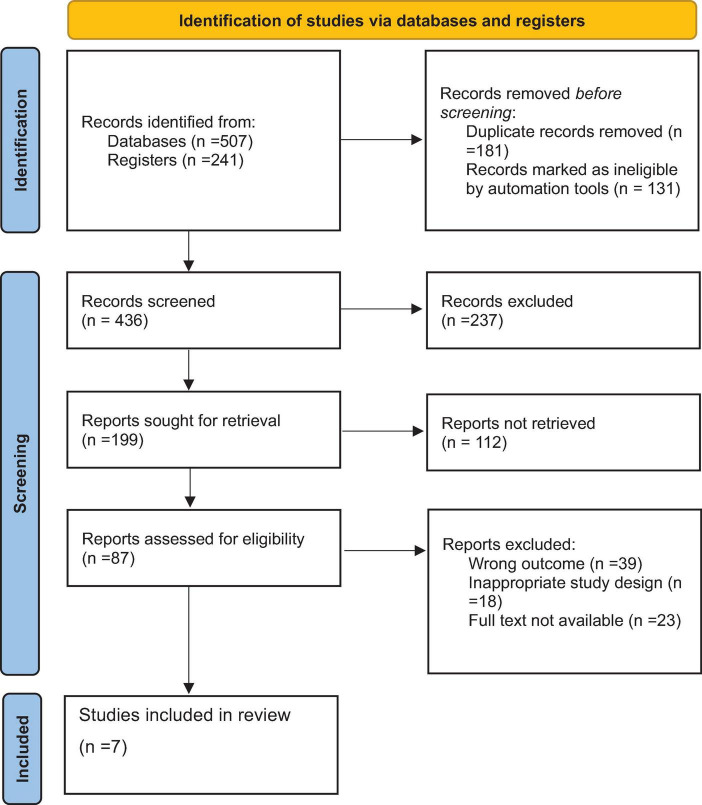
Preferred Reporting Items for Systematic Reviews and Meta-Analyses Protocols (PRISMA) flow diagram of the selection process of studies of prevalence and associated factors of anemia among chronic kidney disease (CKD) patients in Ethiopia, 2024.

After conducting an initial comprehensive search on chronic kidney disease (CKD) research across multiple databases, including Medline, Cochrane Library, Web of Science, Embase, AJOL, Google Scholar, and others, a total of 748 articles were retrieved. Of these, 312 duplicate records were removed. Screening the titles and abstracts of the remaining 436 articles led to the exclusion of an additional 294 articles that did not meet the study criteria. Subsequently, 31 articles were excluded due to unavailability of the full text. Finally, from the remaining 111 articles, seven studies, comprising a total of 1,714 CKD patients, were included in the systematic review and meta-analysis (Figure 1).

### Study characteristics

All seven included studies used a cross-sectional design. Three studies were conducted in Addis Ababa, while each of the remaining four studies was conducted in a different region: one in the Southern region, one in Amhara, one in Oromia, and one in Harar. The reported prevalence of anemia among CKD patients in Ethiopia varied considerably, ranging from 44% to 85.33% (Table 2).

**TABLE 2:** Characteristics of studies included in the systematic review and meta-analysis on prevalence and associated factors of anemia among chronic kidney disease (CKD) patients in Ethiopia, 2024.

Name of author	Year	Region	Health facility name	Study design	Sample size	Anemia among CKD patients
F Bishaw et al.	2023	Oromia	Jimma university hospital	Cross-sectional	150	85.33
B. Alemu et al.	2021	Addis Ababa	Addis Ababa	Cross-sectional	387	53.5
H. Adera et al.	2019	Amhara	Gondar	Cross-sectional	251	64.5
T H Molla et al	2022	Addi Ababa	St Paul hospital	Cross-sectional	352	65.91
A Kidanewold et al	2021	South Ethiopia	south	Cross-sectional	384	44
M U Yusuf et al	2023	Eastern Ethiopia	Harar	Cross-sectional	90	64.8
S Abdi et al	2021	Addis Ababa	Black lion hospital	Cross-sectional	100	48

### Pooled prevalence of anemia among CKD patients in Ethiopia

**FIGURE 2:**
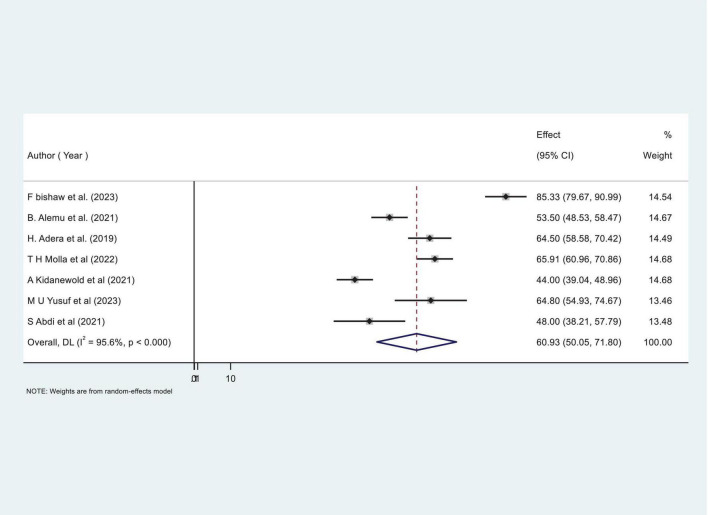
Forest plot showing the pooled proportion prevalence of anemia among chronic kidney disease (CKD) patients in Ethiopia.

Our comprehensive meta-analysis identified a significant anemia prevalence rate of 60.93% (95% CI: 50.05, 71.80) among CKD patients in Ethiopia, with substantial heterogeneity observed across studies (I^2^ = 95.6%, *P* < 0.000), as shown in Figure 2. Due to this high level of variability, we applied a random-effects model, allowing for a robust adjustment to account for the significant heterogeneity among the included studies.

### Subgroup analysis of prevalence of anemia among CKD patients in Ethiopia

**FIGURE 3:**
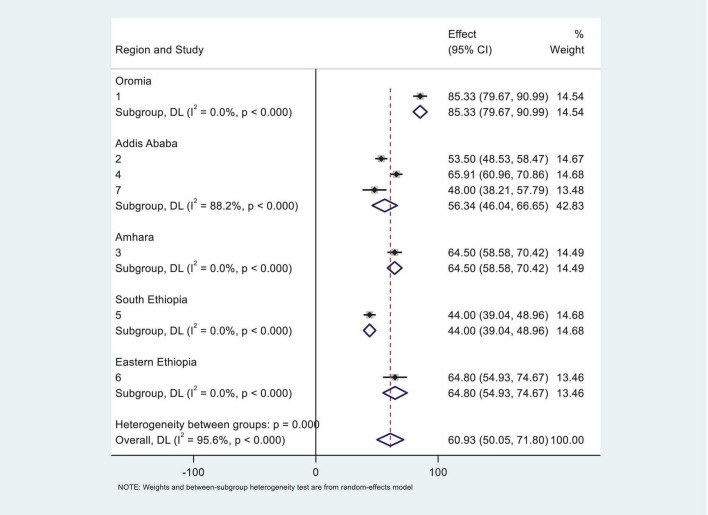
Subgroup analysis of systematic review and meta-analysis by region for the study of prevalence of anemia among chronic kidney disease (CKD) patients in Ethiopia.

To better understand the observed heterogeneity, we performed a subgroup analysis by region, which uncovered striking variations in prevalence rates. Notably, the Oromia region reported the highest prevalence of anemia among CKD patients at an alarming 85.33% (95% CI: 79.67–90.99%). In sharp contrast, the South Ethiopia region exhibited the lowest prevalence, with only 44% (95% CI: 39.04–48.96%) of patients affected. Despite these differences, the overall heterogeneity remained significant, with I^2^ = 97 (Figure 3), underscoring the complexity of anemia prevalence across the country.

### Publication bias and heterogeneity

**FIGURE 4:**
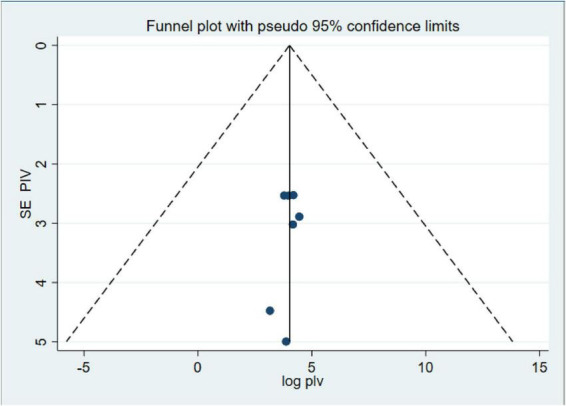
Funnel plot of systematic review and meta-analysis on systematic review and meta-analysis of prevalence and associated factors of anemia among chronic kidney disease (CKD) Patients in Ethiopia, 2024.

Our investigation into publication bias yielded insightful results. The Egger’s test produced a *p*-value of 0.64, indicating no statistically significant evidence of publication bias in our meta-analysis (Table 3). Furthermore, the funnel plot (Figure 4) displayed a symmetrical distribution of the included studies, further confirming the absence of publication bias. These findings bolster the credibility of our results and underscore the robustness of the observed anemia prevalence rates among CKD patients in Ethiopia.

**TABLE 3:** Egger’s test systematic review and meta-analysis of prevalence and associated factors of anemia among chronic kidney disease (CKD) patients in Ethiopia, 2024.

Standard effect	Coefficient	Standard error	t	*P* > t	95% CI
Slope	4.600698	0.5436923	8.46	0.000	5.998303
Bias	–0.1263578	0.1823594	–1.09	0.324	0.2694569

Additionally, to identify potential sources of heterogeneity, we conducted a meta-regression analysis that included sample size and publication year as covariates. However, our findings indicated that neither variable significantly affected the observed heterogeneity across the studies (Table 4).

**TABLE 4:** Meta-regression analysis of factors affecting between-study heterogeneity.

Prevalence	Coeff.	Stad. Err.	t	*P* > ltl	95% conf.	Interval
Sample	–0.0859264	0.0446475	–1.92	0.127	–0.2098877	0.0380348
Year	3.5647	3.690551	0.97	0.389	–6.681913	13.81131
Const	–7118.022	7463.851	–0.95	0.394	–27840.99	13604.95

### Sensitivity analysis

**FIGURE 5:**
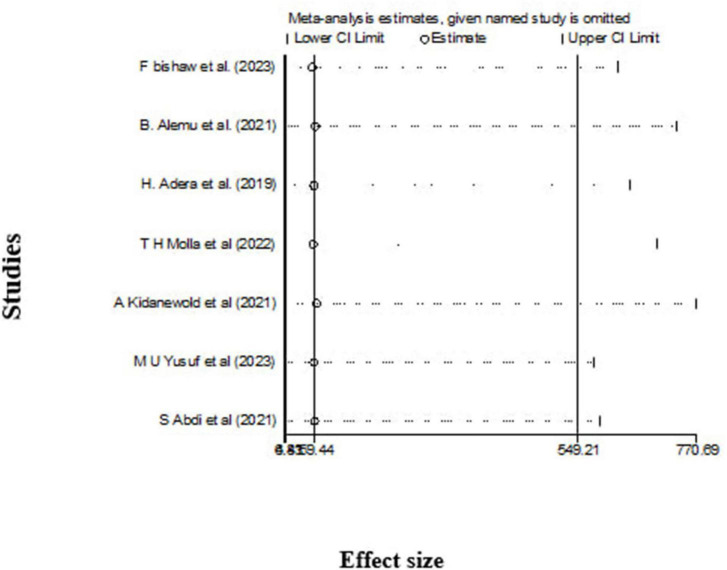
The leave-out-one sensitivity analysis.

The leave-out-one sensitivity analysis was conducted to rigorously assess the impact of individual studies on the overall pooled prevalence of anemia among chronic kidney disease (CKD) patients. In this systematic approach, each study was sequentially excluded from the analysis to evaluate its influence. The results of this thorough evaluation indicated that the exclusion of any single study did not lead to a significant or statistically meaningful change in the overall pooled estimate of anemia prevalence. These findings are illustrated in Figure 5, which demonstrates the stability and robustness of the overall pooled estimate, even with the removal of specific studies from consideration.

### Factors associated with anemia among CKD patients in Ethiopia

**FIGURE 6:**
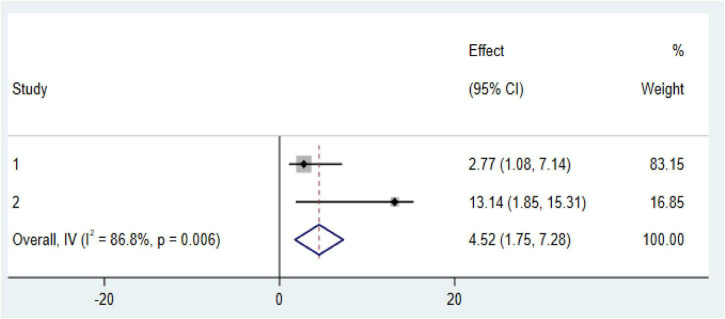
Forest plot of the association between diabetes mellitus (DM) comorbidity with prevalence of anemia among chronic kidney disease (CKD) patients in Ethiopia 2024.

**FIGURE 7:**
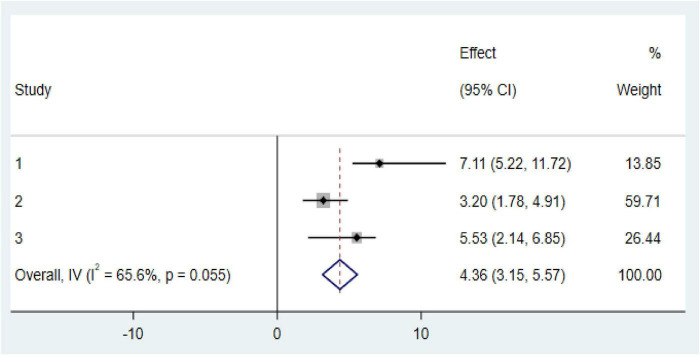
Forest plot of the association between stage 3 and above with prevalence of anemia among chronic kidney disease (CKD) patients in Ethiopia 2024.

Our meta-analysis reveals two significant factors associated with an increased risk of anemia among CKD patients in Ethiopia: diabetes mellitus (DM) comorbidity and advanced CKD stages (stage 3 and above). CKD patients with diabetes comorbidity were found to have markedly higher odds of developing anemia (OR: 4.52, CI: 1.175, 7.28; Figure 6). Similarly, those in stage 3 or more advanced stages of CKD faced an increased risk of anemia (OR: 4.36, CI: 3.15, 5.57; Figure 7).

## Discussion

Anemia in chronic diseases is an emerging public health problem in low-income countries, including Ethiopia. The prevalence of anemia among CKD patients has become a significant burden in developing nations compared to developed countries. This study aimed to provide evidence on the pooled prevalence of anemia and its associated factors among CKD patients in Ethiopia.

In this systematic review and meta-analysis, we summarized the pooled prevalence of anemia among CKD patients in Ethiopia, which was 55.19% (95% CI: 42.19–68.20%). This prevalence is consistent with studies conducted in Turkey (55.9%) (29), Catalonia, Spain (58.5%) (30), Nepal (53.6%) (31), and Sub-Saharan Africa (59.19%) (8). However, the results of this meta-analysis are lower than those from studies conducted in Malaysia (75.8%) (32), Brazil (86%) (33), and Pakistan (80.5%) (34). On the other hand, this finding is higher than results from studies conducted in India (39.36%) (35), Japan (32.3%) (36), and the United States (15.4%) (37). These differences may be attributed to variations in socioeconomic status, study periods, study settings, types of comorbidities and complications, methods for classifying the disease, and the quality of healthcare available to manage CKD patients.

Subgroup analysis in this meta-analysis revealed that the Oromia region reported the highest prevalence of anemia among CKD patients at 85.33% (95% CI: 79.67–90.99%), while the South region showed the lowest prevalence, with only 44% (95% CI: 39.04–48.96%) of patients affected. This inconsistency may be attributed to variations in sampling techniques, sample size, socio-demographic and behavioral characteristics, admission diagnoses, nutritional factors, age of the participants, and clinical complications among the study subjects. Furthermore, studies from other low-income settings, such as South Asia, and Latin America, have reported varying prevalence rates of anemia among CKD patients, which may be influenced by socioeconomic and healthcare disparities (38). Regional variations in anemia prevalence are shaped by cultural, dietary, healthcare, socioeconomic, and environmental factors. Political instability further worsens anemia by disrupting food supply and healthcare. Tailored public health interventions addressing local factors are crucial for reducing anemia prevalence.

Our systematic review and meta-analysis identified significant factors associated with an increased risk of anemia among CKD patients in Ethiopia. In a random-effects model, pooled estimates indicated that diabetes mellitus (DM) comorbidity was significantly associated with anemia among CKD patients; specifically, the pooled estimates showed that the risk of developing anemia was nearly five times higher in CKD patients with DM compared to those without DM. This finding aligns with studies conducted in Sub-Saharan Africa (8), Nepal (39), and the United States (40). This association is primarily due to the effects of chronic hyperglycemia associated with diabetes. Persistent hyperglycemia can create a hypoxic environment in the renal interstitium, leading to impaired erythropoietin production. Additionally, erythrocyte precursor cells in the bone marrow may experience prolonged exposure to glucose toxicity, while mature red blood cells can be damaged by oxidative stress—both of which impair erythropoiesis and reduce red blood cell survival in individuals with sustained hyperglycemia. Other contributing factors to anemia in diabetic patients include chronic inflammation, elevated levels of advanced glycation end products (AGEs), erythropoietin resistance, oxidative stress, and the side effects of anti-diabetic medications (38, 41, 42).

Another important factor associated with anemia among CKD patients was advanced CKD stage (stage 3 and above), which showed a strong association with anemia. The risk of developing anemia was four times higher in CKD patients at stage 3 or beyond. This finding is consistent with previous studies conducted in Pakistan (34), Singapore, Malaysia (43), and the United States (39). These results emphasize the need for targeted interventions to manage anemia in CKD patients, particularly for those with diabetes and those in advanced stages of the disease.

### Strength and limitation of the study

This systematic review and meta-analysis present both strengths and limitations. On the positive side, it offers an updated and comprehensive estimate of anemia prevalence and identified common factors in chronic kidney disease (CKD) patients.

Nevertheless, there are limitations to consider. Although subgroup analysis was performed, it was unable to pinpoint the sources of heterogeneity. The lack of standardized definitions for CKD and varying anemia cutoff points across the studies may have affected the accuracy of the pooled prevalence estimate. Additionally, the findings may not be fully generalizable, as the meta-analysis includes studies from a limited number of regions, which may not represent the broader CKD population. Furthermore, most of the included studies were cross-sectional, preventing the establishment of a cause-and-effect relationship between CKD and anemia. Therefore, further research with stronger study designs, considering additional causes of anemia, is necessary. Finally, the substantial heterogeneity observed among the studies, with unclear origins, makes it difficult to draw definitive conclusions from the results.

## Conclusion

This study found a significantly high pooled prevalence of anemia among CKD patients. Subgroup analysis revealed the Oromia region had the highest anemia prevalence, while the Harar region had the lowest. The study also highlighted that comorbidity with diabetes mellitus and advanced CKD stages (3 and above) were significantly associated with anemia among CKD patients.

### Recommendations

We recommend regular anemia screening in CKD patients, particularly in regions with high prevalence such as Oromia. It is crucial to identify and manage risk factors, including diabetes mellitus and advanced CKD stages, to reduce the incidence of anemia. Additionally, implementing targeted anemia management strategies, such as iron supplementation and erythropoiesis-stimulating agents, will help to mitigate the burden of anemia in CKD. By adopting these recommendations, healthcare systems can improve the prevention and management of anemia in CKD, enhancing patient outcomes and quality of life.

## Data Availability

The data analyzed in this study is subject to the following licenses/restrictions: all datasets used in this analysis are included in the manuscript and are available from the corresponding author upon reasonable request. Requests to access these datasets should be directed to eshetuelf21@gmail.com.
